# General practitioners’ perspectives on barriers to depression care: development and validation of a questionnaire

**DOI:** 10.1186/s12875-020-01224-8

**Published:** 2020-08-01

**Authors:** Arun Senchyna, Milena Abbiati, Juliette Chambe, Dagmar M. Haller, Hubert Maisonneuve

**Affiliations:** 1 Primary Care Unit, Faculty of Medicine, University of Geneva, CMU - 1 rue Michel Servet, CH1211 Geneva, Switzerland; 2 Unit of Development and Research in Medical Education (UDREM), University of Geneva, Geneva, Switzerland; 3 General medicine department, Faculty of Medicine, University of Strasbourg, Strasbourg, France

**Keywords:** Depression, General practitioners, Primary care, Questionnaire

## Abstract

**Background:**

General practitioners (GPs) regularly feel challenged by the care of depressed patients and may encounter several barriers in providing best management. GPs’ perspectives on barriers to depression care are a subject of growing interest but there is a lack of validated assessment tools. The aim of this study was to develop and validate a questionnaire assessing barriers to depression care (BDC-Q) encountered by GPs in France and the French-speaking part of Switzerland.

**Methods:**

The BDC-Q was constructed in five steps: Item development, content validation, pretesting, testing phase and test-retest reliability. The questionnaire items were generated through a literature search. An expert panel of GPs (*n* = 16) and psychiatrists (*n* = 3) validated the content and 20 GPs pretested the questionnaire to provide response process validity evidence. We then tested the questionnaire among 116 GPs and used principal component analysis and internal consistency testing (Cronbach’s alpha) to structure it into consistent dimensions. Test-retest reliability using Pearson correlation coefficient was assessed with 30 GPs who completed the questionnaire twice after an interval of at least 2 weeks.

**Results:**

The 25 items BDC-Q was structured in five dimensions: (i) provision of care by the general practitioner, (ii) considering patients’ attitudes towards depression, (iii) guidance for care, (iv) collaboration with mental health specialists and (v) access to mental health care.

**Conclusions:**

The BDC-Q displays evidence of validity and reliability to meaningfully assess GPs’ perspectives on barriers to depression care. It can be used both at a practice level within a quality improvement strategy, and at a broader level, to inform health planners and tailor appropriate strategies to improve depression care in the community.

## Background

Depression affects more than 300 million people worldwide, accounting now for the first leading cause of disability (WHO). In European countries, the 12 month prevalence of depression is estimated at 6.9% [1]. Along with anxiety disorders, depression is the main mental disorder encountered in primary care (PC) and most depressed patients will be handled in PC settings only [2, 3]. General practitioners (GPs) regularly encounter difficulties to diagnose depression and provide adequate care [4, 5]. Addressing the pitfalls and perspectives for improvements has moved beyond a disease-centred approach to an integrative approach involving the perceptions of patients and care givers [6]. A growing number of studies analyse GPs’ views of their practice and experience to identify barriers to and facilitators of depression care. Authors have described several dimensions of barriers to depression care that involve health care organization, coordination of care, societal influence, patient factors and physician attitudes [7–9]. To date, the current research on perceived barriers to depression care have mostly focused on explorative studies using qualitative designs. These methodological choices have enabled the gain of insight into the complexity of depression care in PC. However, such study designs prevent by nature the generalization of the results. Therefore, quantitative approaches are needed to allow wider assessment of the burden of barriers and explore their relationships and respective weights among communities of GPs. Quantitative assessments of barriers to depression care may be useful to highlight the needs of GPs and in turn, tailor and monitor appropriate responses such as continuous training. Therefore, the aim of the current study was to develop and validate a questionnaire assessing barriers to depression care (BDC-Q) from GPs’ perspectives in order to provide a structured instrument to use in clinical practices.

## Methods

### Design and participants

This is a development and validation study conducted within primary care practices (general internists) in France and the French-speaking part of Switzerland between 2014 and 2018.

**Fig. 1:**
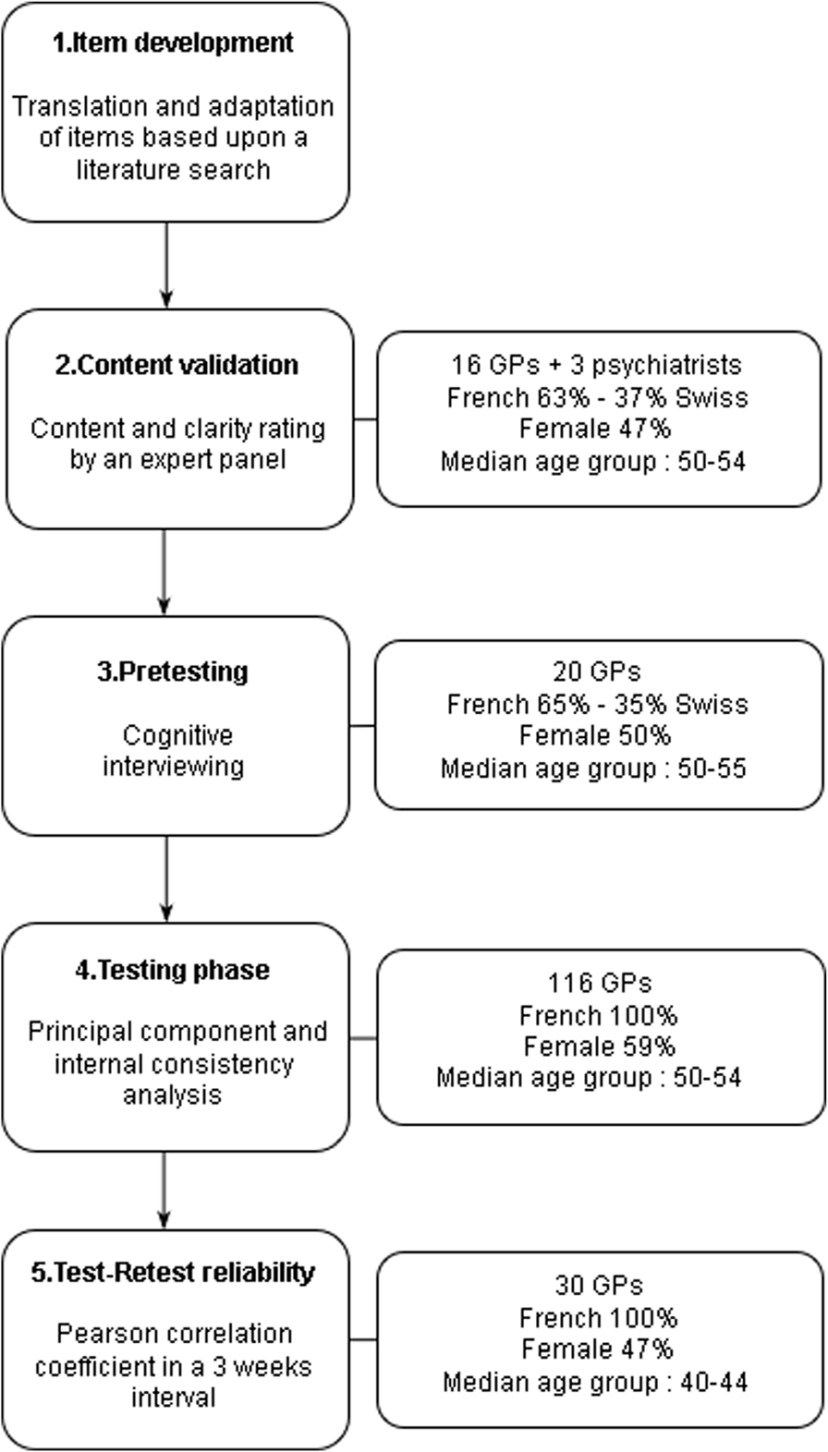
Development and validation steps of the barriers to depression care questionnaire (BDC-Q) with participants’ socio-demographic characteristics

Figure 1 summarizes the development and validation process of the barriers to depression care questionnaire (BDC-Q) with participants’ socio-demographic characteristics.

### Step 1 - item development

We devised items from a literature search conducted in MEDLINE using terms related to depression, family practice, and barriers/facilitators and through bibliographies of retrieved studies. We included qualitative and quantitative studies referring totally or partially to barriers to depression care. We excluded studies that did not involve GPs’ views or were focused exclusively on specific aspects of depression care or populations (e.g. depression in youth, end of life care or postpartum depression). We retained 11 studies from the USA [7, 10, 11], UK [12], France [13], the Netherlands [14], Australia [15, 16], Hong Kong [17] and 2 international meta-syntheses [8, 18]. We also added unpublished results of a focus group previously conducted within a local depression improvement program in the French-speaking part of Switzerland. Two investigators (AS and CC) extracted all elements describing an implicit or explicit barrier. Involving a third investigator (HM), each identified barrier was adapted and translated into French to form an item [19].

### Step 2 - content validation

Content validation was undertaken with a French-speaking panel of 19 physicians with expertise in the management of depression in PC. Physicians were GPs, psychiatrists and academics from Lyon (France), Strasbourg (France) and Geneva (Switzerland). Based on the content validation index described by Polit, experts rated individually through an online survey, the relevance and the clarity of each item on a 4-point scale (not relevant, quite relevant, relevant, highly relevant; respectively: not clear, quite clear, clear, very clear) [20]. An item was considered relevant if more than 75% of experts selected it as “quite relevant” or “highly relevant”. An item was considered clear if it obtained 80% or more of “quite clear” and “clear” ratings [20].

### Step 3 - pretesting

Pretesting of the questionnaire was undertaken to provide response process validity evidence defined as the fit between the construct and the detailed nature of the response engaged in by test takers [21, 22]. We used individual semi-directive cognitive interviewing techniques [23] among 20 GPs who were recruited using snowball sampling for maximal variation [24]. None of the GPs in this step were involved in the content validation step. Two trained investigators (LL and AG) performed the interviews in GPs’ practices. Participants read and laudably answered each item before answering standardised question probes [25]. The probes explored item understanding (e.g. <<In the item “mental health care professionals are available to take on new patients”, what does the word ‘available’ mean to you?>>), redundancy between items, as well as the response selection process [26] (e.g. “How did you decide your answer to this question was strongly agree? ”). All the cognitive interviews were recorded, transcribed verbatim and independently coded by LL and AG using a 12-point coding sheet [25]. For each interview, items were separately coded as “adequate” or alternatively with a combination of 11 issue codes (e.g. “Respondent unsure how to answer since experience varies depending on circumstances”; “Respondent asks for clarification of the item”). An item was judged satisfactory if it was coded “adequate” for more than 85% of respondents [25].

### Steps 4 and 5 - testing phase and test-retest reliability

The testing phase of the BDC-Q was carried out among 985 GPs from the Alsace and Rhone-Alpes regions of France. They were recruited through regional professional organisation mailing lists. Surveys were web-based and gathered socio-demographic characteristics. The questionnaire items were displayed randomly to avoid response contamination bias [27]. We prevented missing data by forcing a response to all items. We used principal component analysis followed by internal consistency testing to organise the items into descriptive dimensions. Test retest reliability was conducted with a convenience sub-sample of 40 GPs in the region of Lyon (France). They were asked to respond to the survey again after a 14-day interval.

### Statistical analysis

We calculated descriptive statistics for each item including the mean, standard deviation, and range to inspect floor and ceiling effects. Items endorsed by more than 95% of the participants were considered for removal [28]. We performed a principal component analysis (PCA) with Promax rotation to aggregate BDC-Q items in factors after using the Kaiser-Meyer-Olkin (KMO) index of sampling adequacy to confirm suitability of the data. We used combined criteria (i.e. eigenvalue > 1 and interpretability) to retain the most relevant factors and order them into consistent dimensions [29]. A minimum factor loading of 0.40 was used as the criterion for each retained item [30]. An item obtaining primary and secondary loading superior to 0.40 was assigned to the dimension with the most theoretical sense. We used classical Cronbach’s Alpha coefficient to assess dimensions’ internal consistency. In doing this our intention was not to create subscales, but just to confirm that the items were sufficiently related, justifying their grouping under the different dimensions. We chose the following critical values for the dimensions internal consistency: α > 0.75 = excellent, α between 0.60 and 0.75 = good, α between 0.40 and < 0.60 = moderate, and α < 0.40 = poor [31]. We used Pearson correlation coefficient to determine dimensions test-retest reliability between time 1 (T1) and time 2 (T2) with the following critical values for Pearson’s *r*: *r* > 0.5 = high, *r* > 0.3 < 0.5 = moderate, and *r* < 0.3 = low [32]. All statistical analyses were conducted using IBM SPSS Statistics version 24.

## Results

### Steps 1 and 2-Item development and content validation

We devised 42 items from the literature and submitted them to the binational expert panelists. Based on the content validation index, 16 irrelevant items were dropped, and 26 items were retained for pretesting with minor modifications to relevant and unclear items (*n* = 5).

### Step 3-Pretesting

Among the 26 items pretested, 13 were adequate and retained without modification. The remaining 13 items were revised in their wording through discussion between the research team members, in accordance with propositions highlighted during the cognitive interviews. Because of two double-barreled items, 2 derived items were added, leading to a 28 items questionnaire. For all items (displayed in Table 1), a traditional 5-point Likert scale was suitable, ranging from strongly disagree (=1) to strongly agree (=5) [19]. The neutral position (=3) was labelled “no answer” to capture a neutral position or a non-existent opinion. In order to obtain a balanced questionnaire, half of the items were positively worded (e.g. “It is easy to distinguish between simple sadness and a depressive disorder”) and the other half negatively (e.g. “Obtaining feedback on patients from mental health care professionals is difficult”).

**Table 1:** Descriptive features of the original items submitted to 116 general practitioners during the testing phase (non-validated English translation of the original items in French)

N°	Item	Mean score (SD; Range)
1	The symptoms of depression are specific	3.2 (1.0; 1–5)
2	It is easy to distinguish between simple sadness and a depressive disorder	2.6 (0.9; 1–5)
3	Screening tools for depression, such as the HAD (Hospital Anxiety and Depression scale) for example, lack practical utility	3.3 (0.8; 1–5)
4	Assessment tools for depression, such as the Hamilton scale or the Beck Depression Inventory lack practical utility	2.6 (1.0; 1–5)
5	Best practice recommendations related to depression lack practical applicability	3.4 (0.9; 1–5)
6	The general public is well informed about depression	2.2 (0.8; 1–4)
7	The general public is well informed about the management of depression	3.9 (0.7; 1–5)
8	Patients suffering from depression endure social stigmatization	3.7 (0.8; 1–5)
9	Patients suffering from depression underestimate the severity of their depression	3.6 (0.9; 2–5)
10	Patients suffering from depression easily accept a diagnosis of depression	2.9 (1.1; 1–5)
11	Patients suffering from depression easily accept being referred to a mental health care professional	2.4 (0.9; 1–5)
12	The commitment of patients suffering from depression to the therapeutic project is limited	3.1 (1.0; 1–5)
13	Patients suffering from depression are adequately reimbursed for their mental health care costs	3.4 (1.1; 1–5)
14	Taking care of a patient suffering from depression often takes up more time than I can give him/her	3.9 (1.1; 1–5)
15	I am adequately paid for taking care of patients suffering from depression	2.2 (1.1; 1–5)
16	Working with patients suffering from depression is heavy	3.4 (1.0; 1–5)
17	Mental health care professionals are available to take on new patients	2.0 (0.9; 1–5)
18	I know the specializations of mental health professionals regarding certain pathologies (for example, addiction, bipolar disorders) well.	2.7 (1.1; 1–5)
19	The capacity of specialized mental health care structures is insufficient	4.0 (0.9; 2–5)
20	I know the services offered by mental health care structures well	2.8 (1.0; 1–5)
21	I mistrust mental health care structures	2.7 (0.9; 1–5)
22	I have had bad experiences using structures specialized in mental health	2.9 (1.0; 1–5)
23	Medical information sharing between patients and mental health care professionals is easy	2.1 (1.0; 1–5)
24	Getting advice over the phone from mental health care professionals is easy	2.2 (1.0; 1–5)
25	Obtaining feedback on patients from mental health care professionals is difficult	4.2 (0.8; 2–5)
26	Expectations concerning the communication of information are the same for general practitioners as for mental health care professionals	2.8 (1.0; 1–5)
27	Setting up meetings with mental health care professionals to discuss cases is difficult	4.0 (0.8; 2–5)
28	The clinical situation of a patient suffering from depression is difficult to summarize in writing	3.3 (1.1; 2–5)

### Steps 4 and 5-Testing phase and reliability

One hundred thirty-one GPs initiated the survey (response rate of 13,3%) with a completion rate of 88,5% (15 incomplete surveys were excluded). Thus, 116 surveys were used for the analysis in the testing phase. Table 1 presents the descriptive statistics for all items. Response means for the 28 items varied from 2.00 to 4.19 with a complete range of responses in 19 out of 25 items (76%) and no floor and ceiling effects observed. Maximum endorsement was 63.8% “disagree” for item 2 (“It is easy to distinguish between simple sadness and a depressive syndrome”).

The PCA, shown in Table 2, yielded five factors (KMO 0.57, *p* < 0.001, 41.4% of variance explained) with the first three factors accounting for 31% of the variance. We interpreted and labelled each factor according to the co-varying items. We named Factor 1 “Provision of care by the general practitioner”, combining items 5; 14; 15; 16; 19; 28. We named Factor 2 “considering patients’ attitudes towards depression”, combining items 2; 6; 9; 10; 11; 12. We named Factor 3 “Guidance for care”, combining items 3; 4; 18; 20. We named Factor 4 “Collaboration with mental health specialists”, combining items 17; 23; 24; 25; 26; 27. We named Factor 5 “Access to mental health care”, combining items 13; 21; 22. We excluded Items 1; 7; 8 since their loadings were < 0.40. Items 15; 17; 24; 28 cross-loaded significantly (> 0.40) on a second Factor but these cross-loadings were always lower than the cross-loadings on their primary Factor, except for item 15. We found a weak correlation between Factor 1 and Factor 3 (0.283).

**Table 2:** Principal Components Analysis with Promax Rotation* of the barriers to depression care questionnaire (BDC-Q)

N° Item (Table 1)	**Factor loadings**				
	F1	F2	F3	F4	F5
14	**.811**	.121	.244	.173	−.098
16	**.680**	.103	−.060	.133	−.173
19	**.488**	.084	.157	.097	.192
28	**.437**	**.415**	.257	.298	−.225
5	**.402**	.072	.212	.130	.094
10 R	.214	**.777**	.227	.020	.060
12	.159	**.601**	−.069	.025	−.307
9	.171	**.562**	−.048	.106	−.125
11 R	−.055	**.557**	.239	.034	.161
6 R	−.073	**.460**	.013	−.031	−.010
2 R	.258	**.408**	.242	.234	−.34
*7 D*	*.052*	*.371*	*.248*	*.153*	*.078*
*8 D*	*.219*	*.230*	*.063*	*.029*	*−.175*
20 R	.235	.022	**.755**	.205	.017
18 R	.176	−.051	**.743**	−.065	−.108
4	.144	.282	**.645**	.108	.082
3	.117	.273	**.544**	.215	−.050
15 R	**.406**	.083	**.420**	−.049	.156
*1 RD*	*.217*	*.287*	*.291*	*.193*	*−.214*
23 R	.007	.139	.038	**.776**	−.031
25	.386	.044	.088	**.635**	.151
17 R	**.430**	−.158	.316	**.486**	.106
27	.376	.032	.276	**.472**	−.012
24 R	.151	−.137	.097	**.459**	**.406**
26 R	−.098	.036	.099	**.454**	.242
21	−.041	−.019	.018	.207	**.677**
22	.031	.024	−.03	.208	**.646**
13 R	.384	.008	.219	−.196	**.554**
	**% Variance explained**				
	F1	F2	F3	F4	F5
	13.906	8.885	6.944	6.255	5.474
	**Factor correlations**				
	F1	F2	F3	F4	F5
F1	1				
F2	.146	1			
F3	.283	.153	1		
F4	.214	.112	.150	1	
F5	.039	− 138	.087	−.012	1

*Kaiser-Meyer-Olkin index = .57, *p* < .001, 41.4% variance explained

*R* reversed, *D* dropped

Table 3 presents the BDC-Q internal consistency (Cronbach’s alpha) and the test–retest reliability (Pearson’s *r*) per dimension. Cronbach’s alpha ranged from 0.48 to 0.69. Scores were good for dimensions 1 to 4 and moderate for dimension 5. Among the 40 GPs who were asked to respond in both instances, 30 (75%) returned the questionnaire within a median T1-T2 interval of 18 days (range 14–55). Pearson correlation coefficient was high for all dimensions, ranging from 0.54 to 0.65 with all *p* < 0.05.

**Table 3:** Internal consistency (Cronbach’s alpha) and Test–retest reliability (Pearson’s r) per dimension of the barriers to depression care questionnaire (BDC-Q)

Factor	Dimension	N° Item (Table 1)	Cronbach’s alpha	Pearson’sr*
			*N* = 116	*N* = 30
F1	Provision of care by the general practitioner	5;14;15;16;19;28	0.610	0.540
F2	Considering patients’ attitudes towards depression	2;6;9;10;11;12	0.639	0.608
F3	Guidance for care	3;4;18;20	0.651	0.631
F4	Collaboration with mental health specialists	17;23;24;25;26;27	0.655	0.653
F5	Access to mental health care	13;21;22	0.477	0.636

*p < 0.05

## Discussion

### Summary of main findings

The five-step validation study led to the BDC-Q with 25 items, covering five dimensions:

(i) provision of care by the general practitioner, (ii) considering patients’ attitudes towards depression, (iii) guidance for care, (iv) collaboration with mental health specialists and (v) access to mental health care. We used a combination of qualitative and quantitative methods in 4 sequential steps to construct the questionnaire and provide evidence of its validity. The BDC-Q is available in French (Additional file 1 – BDC-Q French). We also present an English (unvalidated) translation of the final questionnaire for this publication (Additional file 2 – BDC-Q English).

### Comparison with previous literature

To our knowledge, this is the first study describing the validation of a questionnaire specifically exploring GPs’ views on barriers to depression care. To date, usage of questionnaires to evaluate GPs’ views of depression care has been a subject of growing interest. Haddad et al. recently revised the Depression Attitude Questionnaire (DAQ) which explores professional confidence, therapeutic optimism, and views about generalist or specialist perspectives relevant to depression and its care [33]. Some of the items of the DAQ were included during the development of the BDC-Q but did not come through in the final version, except for a single item (“working with patients suffering from depression is heavy”) which no longer appears in the revised DAQ. We therefore believe that the BDC-Q’s focus is narrower and explores distinct constructs that the DAQ and its revised version do not necessarily capture. In Norway, Bjertnaes et al. validated a questionnaire on GPs’ experience of quality of care in community mental health clinics [34]. We believe that many of the items evoke the dimension “collaboration with mental health specialists” that we present.

The BDC-Q is structured in five original dimensions embracing previous models [7, 8]. The content of the questionnaire is in line with previous studies in France showing that barriers to mental health care involved unsatisfactory co-operation between GPs and mental health services, feelings of stigma and reluctance from patients to consult mental health specialists, as well as high costs of mental health care in the private sector [13, 35, 36].

### Strength and limitations

Preliminary content validation and pretesting steps permitted improvements in terms of relevance and clarity of the items. In turn, the high completion rate during the testing phase suggests that the BDC-Q is acceptable and suitable to administer in everyday practice. Results on the item-level show that there were no remaining floor and ceiling effects, raising the potential to measure cross-sectional differences and responsiveness. This study involved participants and investigators from two countries with distinct health care systems, one with GPs in a gate-keeper role (France) and the other with a more liberal access to primary and specialized care (Switzerland). This somewhat limits the contextual bias. In addition, the items of the BDC-Q were identified within the international literature and not only within the French-speaking context to which participants belong. We therefore believe that the BDC-Q can provide valuable content in other high resource settings. The BDC-Q does not account for depression related to specific health or social conditions. Although different barriers to depression care may be encountered depending on the socio-demographic populations of interest [37], the BDC-Q intends to capture relevant information that is common across different patient groups, and is thus designed for general use. Our study suffers several limitations. First, we devised items through a non-systematic review of the literature. This implies the risk of relevant content loss. However, as we asked for comments, no relevant item was suggested by participants during the validation steps. Second, the testing phase may have suffered from selection bias. Indeed, we are unaware of sociodemographic characteristics and motivations of the respondents as compared to the non- respondents. Respondents in the testing phase may constitute of a subgroup of French GPs expressing more interest and/or difficulties in depression care. Third, results of PCA analysis may be limited by a restricted sample size despite a participant/item ratio of 4, which is common practice in this field [38]. Factorial analysis rather than PCA is preferred to structure a questionnaire into dimensions, but requires a large sample size [39]. Furthermore, Cronbach’s alpha of Factor 5 was weaker. This can be explained by the low number of items (*N* = 3), which mathematically reduces the score compared to factors with a higher number of items [40]. Taking account of these limitations, we do not suggest subscale scoring of the dimensions presented at this stage. The dimensions of the BDC-Q should be considered as indicative and calling for further research, because they may differ in other health care settings or populations of GPs.

### Applications and implications for future research

The validation process of the questionnaire encourages its use to meaningfully assess barriers to depression care perceived by GPs. The BDC-Q can be useful to gathered relevant data at a practice level, within a quality improvement effort. Addressing key barriers is crucial to design adequate interventions which have been shown to be effective in improving mental health care both at the clinical and health policy levels [41]. At the physician level, taking the BDC-Q can raise awareness of common pitfalls in current practices, enabling a better recognition of the resources and limitations encountered while dealing with depressed patients. Similar to a previous study using the DAQ [42], further research could assess whether a relation exists between expressed barriers and actual depression management, for example in terms of diagnosis or treatment choices. A longitudinal research should assess the validity of the BDC-Q to measure change, for example to monitor the impact of targeted training. The BDC-Q may also benefit from translation and cross-cultural validation for use in other settings.

## Conclusion

The BDC-Q displays validity and reliability evidence to meaningfully assess GPs’ perspectives on barriers to depression care in French-speaking settings. By facilitating broad and reliable assessment of such barriers, the BDC-Q can be a useful tool to target key improvement indicators, inform health planners, and tailor appropriate strategies to improve depression care in the community. Further research steps include the translation and validation of the BDC-Q in other languages to allow use in a broader range of primary care settings.

## Supplementary information

**Additional file 1.** Questionnaire sur les Barrières à la Prise en charge de la Dépression en Médecine Générale (BDC-Q). Version française validée.

**Additional file 2.** Questionnaire on Barriers to Depression care in Family Practice (BDC-Q). Unvalidated English version.

## Data Availability

The data that support the findings of this study are available from the authors upon reasonable request.

## Abbreviations

BDC-Q: Barriers to depression care questionnaire
DAQ: Depression Attitude Questionnaire
GPs: General practitioners
KMO: Kaiser-Meyer-Olkin
PC: Primary care
PCA`: Principal component analysis
