# Supplementary material for: General practitioners’ perspectives on barriers to depression care: development and validation of a questionnaire
Source: BMC Fam Pract. 2020 Aug 1;21:156. doi: 10.1186/s12875-020-01224-8 (PMC7395981; doi:10.1186/s12875-020-01224-8)
Supplement: Supplementary file 1 — Additional file 1. Questionnaire sur les Barrières à la Prise en charge de la Dépression en Médecine Générale (BDC-Q). Version française validée. [file 12875_2020_1224_MOESM1_ESM.docx]

**Questionnaire sur les Barrières à la Prise en charge de la Dépression en Médecine Générale (BDC-Q)**

**Version française validée**

Le but de ce questionnaire est d’explorer les barrières à la prise en charge de la dépression en médecine générale. Pour chacun des items suivants, merci de choisir la proposition correspondant le plus à votre expérience personnelle.

|  | **Pas du tout d'accord** | **Pas d'accord** | **Ne se prononce pas** | **D'accord** | **Tout à fait d'accord** |
| --- | --- | --- | --- | --- | --- |
| **La pratique du médecin généraliste :** |  |  |  |  |  |
| 1-Prendre en charge un patient dépressif demande souvent plus de temps que ce que je peux y consacrer |  |  |  |  |  |
| 2- Travailler avec des patients dépressifs est pesant |  |  |  |  |  |
| 3- La capacité d’accueil des structures spécialisées en santé mentale est insuffisante |  |  |  |  |  |
| 4- La situation clinique d’un patient dépressif est difficile à résumer par écrit |  |  |  |  |  |
| 5- Les recommandations de bonnes pratiques concernant la dépression manquent d’applicabilité pratique |  |  |  |  |  |
| 6- Prendre en charge des patients dépressifs est suffisamment rémunéré |  |  |  |  |  |
| **Les patients et les représentations de la dépression :** |  |  |  |  |  |
| 7-Les patients dépressifs acceptent facilement le diagnostic de dépression |  |  |  |  |  |
| 8-L’adhésion des patients dépressifs au projet thérapeutique est limitée |  |  |  |  |  |
| 9-Les patients dépressifs sous-estiment la sévérité de leur dépression |  |  |  |  |  |
| 10-Les patients dépressifs acceptent facilement d’être adressés chez un professionnel en santé mentale |  |  |  |  |  |
| 11-La population générale est bien informée sur la dépression |  |  |  |  |  |
| 12-Il est facile de distinguer une simple tristesse de l’humeur d’un syndrome dépressif |  |  |  |  |  |

|  | **Pas du tout d'accord** | **Pas d'accord** | **Ne se prononce pas** | **D'accord** | **Tout à fait d'accord** |
| --- | --- | --- | --- | --- | --- |
| **Les aides à la prise en charge :** |  |  |  |  |  |
| 13-Je connais bien les services offerts par les structures spécialisées en santé mentale |  |  |  |  |  |
| 14-Je connais bien les spécialisations des professionnels en santé mentale pour certaines pathologie (par exemple : addiction, trouble bipolaire, etc.) |  |  |  |  |  |
| 15-Les outils de dépistage de la dépression, par exemple l’échelle HAD (Hospital Anxiety and Depression scale), manquent d’utilité pratique |  |  |  |  |  |
| 16-Les outils d’évaluation de la dépression, par exemple l’échelle de Hamilton ou l’inventaire de Beck, manquent d’utilité pratique |  |  |  |  |  |
| **La collaboration avec les professionnels en santé mentale (psychiatres, psychologues, infirmières spécialisées, etc.) :** |  |  |  |  |  |
| 17-Le partage d’informations médicales concernant les patients avec les professionnels en santé mentale est facile |  |  |  |  |  |
| 18-Le retour d’informations sur les patients de la part des professionnels en santé mentale est difficile |  |  |  |  |  |
| 19-Les professionnels en santé mentale sont disponibles pour la prise en charge de nouveaux patients |  |  |  |  |  |
| 20-Les rencontres avec les professionnels en santé mentale pour discuter des cas sont difficiles |  |  |  |  |  |
| 21-Obtenir un conseil téléphonique des professionnels en santé mentale est facile |  |  |  |  |  |
| 22-Les attentes concernant la transmission de l’information sont les mêmes entre généralistes et professionnels en santé mentale |  |  |  |  |  |
| **L’accès aux soins en santé mentale :** |  |  |  |  |  |
| 23-Je manque de confiance à l’égard des structures spécialisées en santé mentale |  |  |  |  |  |
| 24-J’ai eu des mauvaises expériences en utilisant les structures spécialisées en santé mentale |  |  |  |  |  |
| 25- Les soins en santé mentale sont bien remboursés aux patients dépressifs |  |  |  |  |  |
