# Supplementary material for: General practitioners’ perspectives on barriers to depression care: development and validation of a questionnaire
Source: BMC Fam Pract. 2020 Aug 1;21:156. doi: 10.1186/s12875-020-01224-8 (PMC7395981; doi:10.1186/s12875-020-01224-8)
Supplement: Supplementary file 2 — Additional file 2. Questionnaire on Barriers to Depression care in Family Practice (BDC-Q). Unvalidated English version. [file 12875_2020_1224_MOESM2_ESM.docx]

**Questionnaire on Barriers to Depression care in Family Practice (BDC-Q)**

**Unvalidated English version**

The aim of this questionnaire is to explore barriers to depression care in family practice.

For each of the following item, please choose the answer that best corresponds to your own experience.

|  | **Strongly disagree** | **Disagree** | **No answer** | **Agree** | **Strongly agree** |
| --- | --- | --- | --- | --- | --- |
| **Provision of care by the family practitioner:** |  |  |  |  |  |
| 1-Taking care of a patient suffering from depression often takes up more time than I can give him/her |  |  |  |  |  |
| 2-Working with patients suffering from depression is heavy |  |  |  |  |  |
| 3-The capacity of specialized mental health care structures is insufficient |  |  |  |  |  |
| 4-The clinical situation of a patient suffering from depression is difficult  to summarize in writing |  |  |  |  |  |
| 5-Best practice recommendations related to depression lack practical applicability |  |  |  |  |  |
| 6- I am adequately paid for taking care of patients suffering from depression |  |  |  |  |  |
| **Considering patients’ attitudes towards depression:** |  |  |  |  |  |
| 7-Patients suffering from depression easily accept a diagnosis of depression |  |  |  |  |  |
| 8-The commitment of patients suffering from depression to the therapeutic project is limited |  |  |  |  |  |
| 9-Patients suffering from depression underestimate the severity of their depression |  |  |  |  |  |
| 10-Patients suffering from depression easily accept being referred to a mental health care professional |  |  |  |  |  |
| 11-The general public is well informed about depression |  |  |  |  |  |
| 12- It is easy to distinguish between simple sadness and a depressive disorder |  |  |  |  |  |

|  | **Strongly disagree** | **Disagree** | **No answer** | **Agree** | **Strongly agree** |
| --- | --- | --- | --- | --- | --- |
| **Guidance for care:** |  |  |  |  |  |
| 13- I know the services offered by mental health care structures well |  |  |  |  |  |
| 14- I know the specializations of mental health professionals regarding certain pathologies (for example, addiction, bipolar disorders) well |  |  |  |  |  |
| 15-Screening tools for depression, such as the HAD (Hospital Anxiety and Depression scale) for example, lack practical utility |  |  |  |  |  |
| 16-Assessement tools for depression, such as the Hamilton Scale or the Beck Depression Inventory lack practical utility |  |  |  |  |  |
| **Collaboration with mental health specialists (psychiatrists, psychologists, specialized nurses, etc.):** |  |  |  |  |  |
| 17-Medical information sharing between patients and mental health care professionals is easy |  |  |  |  |  |
| 18-Obtaining feedback on patients from mental health care professionals is difficult |  |  |  |  |  |
| 19-Mental health care professionals are available to take on new patients |  |  |  |  |  |
| 20-Setting up meetings with mental health care professionals to discuss cases is difficult |  |  |  |  |  |
| 21-Getting advice over the phone from mental health care professionals is easy |  |  |  |  |  |
| 22-Expectations concerning the communication of information are the same for general practitioners as for mental health care professionals |  |  |  |  |  |
| **Access to mental health care:** |  |  |  |  |  |
| 23- I mistrust mental health care structures |  |  |  |  |  |
| 24- I have had bad experiences using structures specialized in mental health |  |  |  |  |  |
| 25-Patients suffering from depression are adequately reimbursed for their mental health care costs |  |  |  |  |  |
